# The safety profile of favipiravir should not be the first argument to suspend its evaluation in viral hemorrhagic fevers

**DOI:** 10.1371/journal.pntd.0008259

**Published:** 2020-06-25

**Authors:** Denis Malvy, Anne-Marie Taburet, Xavier de Lamballerie, France Mentre, Fabrice Extramiana

**Affiliations:** 1 Inserm 1219, University of Bordeaux, Bordeaux, France; 2 Department for Infectious and Tropical Diseases, University Hospital Center of Bordeaux, Bordeaux, France; 3 UMR1184, Inserm, CEA, University Paris Saclay, Le Kremlin-Bicêtre, France; 4 IRD 190, Inserm1207, University Aix-Marseille, Marseille, France; 5 IAME, Inserm, University of Paris, Paris, France; 6 CNMR, Maladies Cardiaques Héréditaires Rares & Department of Cardiology, AP-HP, Université de Paris, Paris, France; University of Texas Medical Branch / Galveston National Laboratory, UNITED STATES

Favipiravir (Toyama Chemical Co., Ltd., Japan) is a small antiviral compound that acts as a direct nucleobase analogue targeting the viral polymerase complex machinery of many RNA viruses with a capacity for an error catastrophe induction resulting into lethal mutagenesis and for chain termination. The drug is approved in Japan for use in severe or pandemic influenza. Besides, it was considered as a promising candidate for investigational repurposed therapy during the 2013 to 2016 West African Ebola outbreak. Favipiravir has not unequivocally proved successful in the specific treatment of Ebola virus disease, even though one Phase II trial could not exclude beneficial effects in mild to moderate cases, but target plasma concentrations were not achieved [1,2].

Recently, the safety of the drug was questioned from a single case report in a patient with severe Ebola virus disease [3]. This patient was treated with oral favipiravir using the regimen investigated in the setting of the Phase II trial and the monitored emergency use program conducted in Guinea in 2014 and 2015 (6,000 mg on the first day and 1,200 mg twice daily on the following 9 days). In this patient, the authors reported a heart rate corrected QT interval (QTc) prolongation close to 100 milliseconds observed on the final 10th day of administration and was subsequently resolved following the interruption of exposure to the drug. The issue is of concern, as a drug-induced QTc prolongation above 60 milliseconds is considered as associated with a high risk of torsade de pointes potentially leading to life-threatening ventricular fibrillation [4]. It is hence critical to rule out other potential causes for QTc prolongation before ascertaining the relationship between the observed QTc prolongation and favipiravir intake. As pointed out in the article, several medications were concomitantly administered to the patient, their effect on ventricular repolarization and QTc interval are listed below. Levofloxacin, a fluoroquinolone class agent blocks the hERG channel (IKr) thereby prolonging the QTc interval [5]. Levofloxacin is listed as a drug with a “known risk” of torsade de pointes in CredibleMeds [6]. Although, the effect of levofloxacin and ceftriaxone has been ruled out in the original paper due to the unmatched timeline of administration, drug to drug interactions involving several drugs are complex and, as suggested by the authors, tissue accumulation may have to be taken into account. The antimalarial mefloquine is also a potassium channel (IKs) blocker and although it does not seem to prolong the QT interval duration alone, it has been shown to induce a synergistic QT prolongation when coadministered with an IKr blocker [7]. Ceftriaxone does not prolong QTc interval when taken alone, but its combination with lanzoprazole has also been reported to induce QTc lengthening [8]. Noteworthy, the patient described also took a proton pump inhibitor therapy (omeprazole) in association with ceftriaxone. A drug to drug interaction between ceftriaxone and omeprazole might warrant further evaluation. Both furosemide and omeprazole may change potassium and magnesium levels and are both listed as drugs with a “conditional risk” of torsade de pointes [6].

In addition to drug related changes in QTc interval duration, other modulating factors should not be overlooked. The electrocardiogram in the Fig 2 of the case report displays profound changes in T-wave morphology [3]. Such changes may be observed after myocardial ischemia, during a Takotsubo episode but also in patients with myocarditis. The presence of a pericardial effusion suggests the presence of an inflammation process at least in the pericardium but also possibly at the myocardium level. In this context of systemic viral infection with tissue pathogenicity and host-driven cell destruction, it would have been important to document or rule out a myopericarditis (by magnetic resonance imaging and cardiac troponin assay).

Therefore, favipiravir definitely cannot be considered as the unique causative factor for the observed QTc prolongation in the case report, particularly since no plasma concentration of the drug was available or produced.

On this behalf, the issue of cardiotoxicity regarding a potential risk of QT/QTc interval prolongation and consecutive fatal cardiac dysrhythmia has been early addressed in numerous preclinical and clinical toxicology studies that were conducted by the manufacturer marketing favipiravir in the context of obtaining its homologation in Japan. Among others, the effect of favipiravir on QT/QTc interval duration has been tested in a thorough QT study using a randomized, double-blind, 4-group, 4-period crossover, placebo- and positive-controlled design. The study was able to detect moxifloxacin-induced QT prolongation (i.e., the positive control), but no significant effect was observed 6 hours after administration of 2,400 mg of favipiravir (ΔΔQTcF = 0.5 ms; 90% confidence interval −1.88 to 2.88; ΔΔQTcF refers to placebo-adjusted, change-from-baseline of QTcF; QTcF refers to corrected QT by Fridericia correction formula). In addition, no dose-response effect on QTc interval was observed with favipiravir [9].

Conversely, favipiravir has also been investigated in Phase III trials implemented in the US [10] and had also been used in the recent epidemic of Ebola virus disease in West Africa using a much higher dosage than the one used for treating influenza virus infections. In the setting of the pioneer Phase II JIKI trial, 126 patients with acute Ebola virus disease were recruited in Guinea from late 2014 until April 2015 [1]. Outside the JIKI trial, favipiravir treatment was offered in the Coyah Ebola treatment center in coastal Guinea by February 2015 to 81 patients with confirmed Ebola virus disease on a monitored emergency compassionate-use basis, using the same dosage and procedures as the JIKI trial [11]. Consecutively, six patients who contracted Ebola virus disease in March 2015 in coastal Guinea were included in the PREVAIL II trial that was planned to evaluate the activity of the antibody cocktail ZMapp. They were concurrently managed with a regimen of favipiravir as part of the standard of care, which was administered at the same dosage and duration than the intervention conducted in the Phase II trial [12].

More noticeably, two convalescent male patients who recovered from acute Ebola disease but carried Ebola virus RNA in semen were enrolled in the FORCE trial (ClinicalTrials.gov. NCT02739477) that has been launched in Guinea in April 2016 to assess the tolerance and activity of higher doses of favipiravir for a longer duration. They were administered favipiravir with a loading dose of 2,400 mg twice a day, followed by a maintenance dose of 1,800 mg twice a day for a total of 14 days, with close daily clinical and electrocardiogram monitoring (unpublished data). In addition, two patients with severe Ebola virus disease, who were part of the cluster of postepidemic resurgence of Ebola virus disease in forest Guinea that occurred in March and April 2016 [13] were treated on an emergency compassionate-basis by a combination of the antibody cocktail ZMapp with oral-administered favipiravir with a loading dose of 6,000 mg on day 1, followed by a maintenance dose of 2,400 mg twice a day in an attempt to reach higher exposure than the one observed in the JIKI trial [2]. Specific treatment with favipiravir was maintained until virological remission and before discharge, for a total of 15 days for patient 1 and 10 days for patient 2 (unpublished data). From this experience with high doses, no cardiac liability with favipiravir was reported, even if no electro cardiac parameters were systematically available in the context of administration for acute Ebola virus disease in the field.

In conclusion, the case report underlines the concomitant prescription of favipiravir and other drugs with QTc prolongation potential. For this reason and also because of potential unknown drug to drug interaction, it seems reasonable to monitor the electrocardiogram and the QTc interval duration in patients treated with high doses of favipiravir and several other drugs. However, although this case report describing multiple causes for QTc prolongation should draw our attention to the occurrence of a rare adverse event, it should not prevent evaluating a drug with potential interest for the treatment of several epidemic prone diseases that can result in a viral hemorrhagic fever syndrome, including ebolavirus and Lassa fever virus infections. At this point, although two specific antibody therapies for Ebola virus disease have performed well in a late-stage clinical trial being conducted during the 2018 to 2019 Ebola outbreak in the Democratic Republic of the Congo [14], trials should still consider investigational agents like other direct antiviral candidate that can target Ebola virus in the brain while harboring the capacity to cross the blood–brain barrier and reach all sites thought to be the reservoirs of Ebola virus in humans.

Finally, further efforts should be provided to better assess the tolerance and activity of high-dose favipiravir, given its ease of oral administration especially adapted to the management of high-risk contact persons of patients infected by susceptible viruses for which no vaccine is still available.
